# The effect of heavy-resistance core strength training on upper-body strength and power performance in national-level junior athletes–a pilot study

**DOI:** 10.3389/fphys.2025.1617104

**Published:** 2025-06-11

**Authors:** Atle Hole Saeterbakken, Tor Einar Sandvikmoen, Erik Iversen, Thomas Bjørnsen, Nicolay Stien, Vidar Andersen, Suzanne Scott, Olaf Prieske

**Affiliations:** ^1^ Department of Sport, Food and Natural Sciences, Faculty of Education, Arts and Sports, Western Norway University of Applied Sciences, Sogndal, Norway; ^2^ Fysioterapeut Tor Einar Sandvikmoen, Helsehuset Kristiansand, Kristiansand, Norway; ^3^ Fysioterapeut Eirk Iversen, Kristiansand Fysioterapi, Kristiansand, Norway; ^4^ Olympiatoppen SØR, Kristiansand, Norway; ^5^ Department of Education and Sports Science, Faculty of Arts and Education, University of Stavanger, Stavanger, Norway; ^6^ School of Anatomy, University of Bristol, Bristol, United Kingdom; ^7^ Division of Exercise and Movement, University Applied Sciences for Sport and Management Potsdam, Potsdam, Germany

**Keywords:** core capacity, trunk, swimming, kayak sprint, power, isokinetic

## Abstract

**Introduction:**

The concept of core strength refers to the ability of the core muscles to transfer, absorb and re-direct energy, and generate force/torque while providing proximal stability for distal mobility of the limbs. The aim of the present study was to examine the effects of an 8-week heavy-resistance core strength training (HR-CST) program on upper-body strength and power performance in young athletes. The secondary aim was to examine the role of sport-specific training background (kayak sprinters vs. swimmers) and sex (males vs. females).

**Methods:**

Eighteen national-level junior athletes (age: 17.1 ± 1.1 years, body height: 178 ± 7.8 cm, body mass: 70.2 ± 10.4 kg, 12 males, 6 females) competing in kayak sprint (n = 6) and swimming (n = 12) volunteered to participate. During the 8-week intervention period, half (i.e., 45 min) of the regular strength training program was replaced with HR-CST. Pre and post intervention, upper-body strength and power (i.e., maximal isokinetic stroke force [MIF] and power [MIP]) were tested by means of a maximal stroke test using a paddle ergometer. Additionally, peak (PP_20_) and average power (AP_20_) was determined in a 20-s all-out stroke test.

**Results:**

Paired sample t-tests indicated that PP_20_ and AP_20_ were significantly improved by 12.8% (p < 0.001, ES = 0.30) and 11.9% (p < 0.001, ES = 0.28), respectively, following HR-CST. No statistical changes were observed in MIF and MIP (p > 0.05, 0.19 ≤ ES ≤ 0.63).

**Conclusion:**

8 weeks of HR-CST appears to be an effective means to improve upper-body strength and power performance in national-level junior kayak sprinters and swimmers. Our results suggest that a dynamic high-intensity core strength-training is a viable option for improving their performance in a periodized pre-season program and should be considered.

## Introduction

The anterior trunk and the lumbopelvic-hip complex, often referred to as the core, connect the lower- and upper extremities and include the spine, hips and pelvis, abdominal structures and the proximal lower limb (Bergmark, 1989; Kibler et al., 2006). The core muscles, and the superficial global muscles connecting upper and lower limbs to the trunk (e.g., gluteus maximus, latissimus dorsi, pectoralis major) contribute proximal stability, enabling distal limb segments to generate range of movement and transfer force and torque across the trunk and between limbs (Hibbs et al., 2008; Willardson, 2007). The term “core training” has been widely used to describe exercises focusing on proximal stability including the muscles which attach to and connect to the spine, pelvis, and hips (i.e., the core muscles) (Hibbs et al., 2008; Akuthota and Nadler, 2004; Borghuis et al., 2008). According to Bergmark (1989), global core muscles (internal and external obliques and rectus abdominis) are responsible for the transfer of force and torques from the trunk to the limbs whereas the local deeper lying core muscles (spinal multifidi and transversus abdominis muscles) stabilize the trunk and spinal column. Typically, core training protocols can be described as low intensity, high volume programs with predominantly isometric exercises aiming to isolate specific core muscles (e.g., the plank) (Dale and Lawrence, 2005; Stephenson and Swank, 2004; Reed et al., 2012).

The core plays a crucial role in executing and acquiring sport-specific skills due to its capacity to transfer, absorb, re-direct energy, and generate/relay force and torque (Kibler et al., 2006; Hibbs et al., 2008; Borghuis et al., 2008; Reed et al., 2012). Specialized core training protocols (i.e., core stability training, core strength training) have been applied for rehabilitation, injury prevention and sport performance (Willardson, 2007; Stuber et al., 2014; Chang et al., 2015). The importance of core strength/stability has been described for athlete enhancement in sport activities and performance (Kibler et al., 2006; Hibbs et al., 2008; Prieske et al., 2016a; Rodríguez-Perea et al., 2023). During the last decade, different core training strategies and studies have included competitive athletes (Hibbs et al., 2008; Reed et al., 2012; Saeterbakken et al., 2022). Moreover, meta-analyses have demonstrated moderate effects in favor of core strength training on sport specific performance, when compared to controlled conditions (Rodríguez-Perea et al., 2023; Saeterbakken et al., 2022; Prieske et al., 2016b), although several studies adopted training protocols associated with rehabilitation settings (Stuber et al., 2014; Chang et al., 2015). For instance, Stanton et al. (2004) and Tse et al. (2005) included low-intensity core strength/stability training and did not find improvement in running (Stanton et al., 2004) or rowing performance (Tse et al., 2005) in athletes. In contrast, low-repetition, high-intensity dynamic core strengthening interventions have shown significant improvements in throwing velocity in young female handball players (Saeterbakken et al., 2011) and drive distance in male elite golfers (Sung et al., 2016). It could be speculated that these contrasting findings are a result of different core training approaches (Saeterbakken et al., 2022). Furthermore, most of the scientific literature on core training include males, or a population from a specific sport background (e.g., handball, soccer, swimming) (Saeterbakken et al., 2022; Prieske et al., 2016b), therefore little is known about the effects of biological sex and sports background on core training response.

In this regard, swimmers and kayak sprinters require a strong and stable core. For example, swimmers must maintain body alignment in the water, while maximizing stroke force and power of upper- and lower limbs (McGill, 2010; Ji et al., 2021). In a paddling stroke, the core muscles stabilize the upper body promoting maximal stroke force generation from a constrained (i.e., small base of support), seated posture on an unstable surface (Alvarez-Yates et al., 2024). Furthermore, Fry and Morton (Fry and Morton, 1991) demonstrated medium-sized correlations between peak isokinetic trunk rotation torque during paddle strokes and kayak sprint performance in internationally ranked kayak sprinters. In swimming, several studies have examined core training and performance and reported mixed results (Scibek, 1999; Karpinski et al., 2020; Patil et al., 2014; Ika et al., 2022; Weston et al., 2015). More specifically, Karpiński and colleagues (Karpinski et al., 2020) observed an improvement in 50m crawl performance after 6-week high-intensity core training in junior elite swimmers, while Scibek (1999) reported improvement in core stability without beneficial transfer effects to swimming performance. Furthermore, Zinke et al. (2019) recruited world-class kayak sprinters to an 8-week isokinetic trunk rotation resistance training intervention and demonstrated significant improvements in peak trunk rotation torque.

Still, and to the best of the authors’ knowledge, no core training intervention in elite athletes has included dynamic and unilateral exercises at high loads (i.e., intensity) and few repetitions (i.e., volume) with the goal of increasing core capacity in athletes. Of note, progressive high-intensity core strength training in previous studies in sports such as handball, golf and swimming (Saeterbakken et al., 2011; Sung et al., 2016; Karpinski et al., 2020; Weston et al., 2015; Dahl and van den Tillaar, 2021) confirmed improvements in sport-specific performance. In contrast, core strength training programs using isometric, low intensity, stability and core exercises did not reveal any gains in sports-specific performance (Stanton et al., 2004; Tse et al., 2005; Schibek, 1999). Therefore, the aim of the present study was to examine the effect of an 8-week heavy-resistance core strength training (HR-CST) program on maximal upper-body strength and power in (national-level junior) athletes. The secondary aim was to examine whether there was an effect of biological sex and sport-specific training history on responses to HR-CST induced effects in kayak sprinters vs swimmers. With reference to the literature (Saeterbakken et al., 2011; Sung et al., 2016; Karpinski et al., 2020), we hypothesized that an 8-week HR-CST program would significantly improve maximal isokinetic stroke force (MIF), maximal isokinetic power output (MIP), and 20-s all-out stroke performance (AP_20_, PP_20_) in national-level junior athletes.

## Methods

### Design

The present investigation was an uncontrolled experimental pilot study using a single-group, repeated measures design. The intervention comprised 8 weeks of HR-CST and was conducted in a sample of young athletes. The intervention took place during the pre-season/preparation phase (i.e., the spring) for both the kayak sprinters and swimmers, and HR-CST sessions were integrated into the regular resistance training (RT) program by replacing half (i.e., 45 min) of the athletes’ weekly RT twice per week (90 min HR-CST per week). All participants were tested before and after the intervention period, using a maximal and a 20-s all-out stroke test to assess upper-body strength and power measures. Due to pre-season time constraints, it was not possible to familiarize participants prior to testing. Nevertheless, the athletes trained using the seated cable row or standing bent-over row on a regular bases, which mimic a kayak stroke.

### Participants

In the present pilot study, 19 highly trained junior athletes competing at national level in kayak sprinting and swimming were recruited. Both swimmers and kayakers were included, as they perform unilateral upper-body actions with rotational movements of the trunk. The participants (age range 16–19 years) included 12 males and 7 females competing in kayak sprinting (i.e., 4 males and 3 females) and swimming (i.e., 8 males and 4 females). No control group (passive or active) was included, as we could not expect athletes to stop training for 8 weeks and the total number of available athletes in the region was limited. To be included, participants had to be at national level (McKay et al., 2022), free of injuries, show a minimum attendance rate of 80% at the pre-planned HR-CST sessions, and participate voluntarily. In total, participants had won more than 20 junior national championships titles in their respective sports. Athletes competed at sprint distances with kayakers participating in 200-m and 500-m competitions, and swimmers competing at 50–200 m distances (including front and back crawl, butterfly, and breaststroke). One female kayak sprinter withdrew from the study, due to low motivation to continue training. In total, 18 athletes (mean age: 17.1 ± 1.1 years, body height: 178 ± 7.8 cm, body mass: 70.2 ± 10.4 kg) completed the study and were included in further analysis. All participants were informed of the possible risk of participating and gave written consent before being enrolled in the study. The study was approved by the Norwegian Centre of Research data (ref nr: 788,668) was conducted according to the national laws and regulations, the University`s ethical guidelines, and the latest version of the Helsinki declaration.

### Assessment of upper-body strength and power

#### Maximal stroke test

**FIGURE 1:**
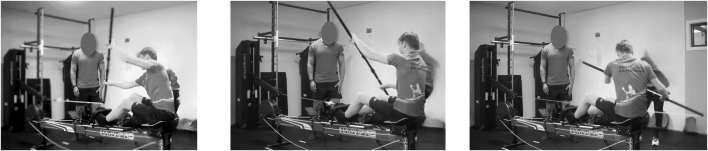
The set-up for the isokinetic testing of maximal stroke force and power.

For the assessment of upper-body strength and power, a maximal stroke test was conducted using a paddle ergometer test (Speedstroke, Kayakpro, New York, USA) (Figure 1). The testing procedures started with a 10-min, progressive warm-up. The distance between the feet rest and chair was adjusted so that knee flexion was approximately 20° (with 0° corresponding to fully extended legs), Grip width of the paddling oar was individually selected. Both distances (i.e., feet-to-seat and grip width) were recorded and used at post-test. For the swimmers, a brief instruction (5–10 min) was conducted by the kayak sprint coach to make sure they sat comfortably and performed the technique properly. All kayak athletes were accustomed to the paddling ergometer from their dry-land training routines.

The warm-up started with 2-min` easy paddling with a gradual increase in trunk rotation and reduction of elbow flexion. After that, the intensity (i.e., stroke frequency) gradually increased across three intervals lasting three, two, and 1 minute, to prepare the participants for maximal effort. No objective measurement was used to control the warm-up intensity, but each participant was informed about the procedures and increased their effort at each interval. Each interval was separated by a 1-min rest, with a 5-min rest interval provided prior to maximal testing.

To measure average and peak maximal isokinetic stroke force (MIF) and power (MIP), the end of the paddle oar was attached via a cable to a computerized robotic dynamometer system (1080 Quantum synchro, 1080 Motion AB, Stockholm, Sweden, recording frequency of 333 Hz). Due to the dynamometer’s construction, it was possible to measure isokinetic force output only on one side at a time. Participants simulated the starting position in kayak sprinting (i.e., the oar facing forward and downwards on one side) and performed four consecutive strokes on the test side using maximal effort (Figure 1). Verbal encouragement was given during each test trial. Maximal concentric stroke force and power were tested at two movement velocities starting at 0.8 m/s followed by 1.5 m/s (Alvarez-Yates et al., 2024). The eccentric phase was always isotonic at a load of 49 N, with a movement velocity of 6.0 m/s. Participants were instructed to follow the eccentric phase of the paddle stroke in a controlled manner. A short rest (≤0.5 s) separated each stroke. A 1-min rest separated the two isokinetic speeds, as well as test side and between test trials on the contralateral side, which was conducted using identical procedures. The average of the last two strokes was used for further analysis. The intraclass correlation coefficient (ICC) between the two strokes demonstrated excellent reliability for the 0.8 m/s (ICC = 0.944–0.997) and 1.5 m/s conditions (ICC = 0.902–9.995) (Langer et al., 2023).

#### 20-s all-out stroke test

After a 5 minute pause, a 20-s all-out kayak sprint test was conducted on a paddle ergometer (Pro Kayak, Dansprint, Hvidovre, Denmark). The air resistance was set to maximum (i.e., resistance 10). Maximum resistance in the paddle ergometer mimics the resistance in a paddling stroke in water and the 20-s duration corresponds to previous protocols (Valiulin et al., 2022). Set up position during the test Was identical to the maximal stroke test. After the participants reached a steady paddling level of 70 W, the test leader initiated a 10-s countdown, after which participants performed a 20-s all-out sprint from which the peak (PP_20_) and average power (AP_20_) were recorded.

### Training protocol

The participants’ weekly training routines included two RT sessions of 90 min. During the intervention period, 45 min of each RT session were replaced by HR-CST. In general, the athletes performed the same strengthening exercises used in their regular program (i.e., squats, pullups, clean, bench press, arm and shoulder exercises), but reduced the overall volume to complete them in 45 min. Before regular RT, HR-CST was instructed and supervised by two strength and conditioning coaches. The core training program was carried out in groups of six individuals, due to limitations of space and available equipment. Each HR-CST session consisted of four to five warm-up exercises consisting of 10–12 repetitions and a single set of each exercise. The warm-up included balance exercises on a Swiss ball, requiring slow and controlled movements, using bodyweight and dynamic stabilization. The main session consisted of four to six exercises applied as a circuit, with heavy loads and 5–10 (avg.: 7–8) repetitions, depending on individual athlete response, and with two sets of each exercise. Each set was performed to failure, meaning the athletes could not complete a further repetition while maintaining a stable core position (Kibler et al., 2006; Saeterbakken et al., 2011; Saeterba et al., 2018). In each session, at least two of the four to six exercises imposed an unilateral pattern (i.e., one arm or one leg only) with a rotational component.

The HR-CST program consisted of exercises designed to improve core strength/stability. Intensity and complexity, in terms of the demands on balance and coordination,were increased in week three and week five. Week one and two included a familiarization to the exercises to make sure movements were executed correctly. In weeks three and four intensity was increased whereas new exercises were introduced in week five (See Supplementary Material for more details). During HR-CST, participants continued their regular training routine, which consisted of four to five weekly sessions of swimming or kayaking, respectively, in addition to the two weekly strength training sessions. The participants were instructed to avoid high intensity training 48 h before pre- and post-intervention testing.

## Statistics

Visual inspection of the QQ-plots and the Shapiro-Wilk test demonstrated normally distributed data (P = 0.088–0.978). All data were presented as the mean ± standard deviation. Pre- and post-intervention test performance for the whole group of athletes was compared using dependent T-tests. To examine the potential effect of biological sex and sports specific training history on percentage change in post-test sprint performance, independent T-tests were used to compare between athlete groups. In order to account for multiple comparisons and an alpha inflation error, a Bonferroni post-hoc correction was applied (Myraunet et al., 2025). The alpha level was set at 0.05 for statistical significance. Cohen`s d effect size (ES) was calculated as a measure of practical concern. An ES of <0.2 was considered trivial, 0.2 to <0.5 as small, 0.5 to <0.8 as moderate, and >0.8 as large (Cohen, 1988). SPSS statistical software (IBM Corp. Released 2020. IBM SPSS Statistics for Windows, Version 28.0. Armonk, NY: IBM Corp) was used for all analyses.

## Results

At baseline, no significant differences were observed between the kayak sprinters and swimmers in the maximal stroke tests (P = 0.734–0.899) or the 20-s all-out stroke test measures (P = 0.459 and 0.548). However, males demonstrated a significantly larger performance output compared to females in all test outcomes (P < 0.001). Performance characteristics of kayak sprinters and swimmers are presented in Tables 1, 2.

**TABLE 1:** Overview of maximal stroke force and power for the kayak sprinters (n = 6).

Test	Condition	Pre	Post	Mean difference (95% CI)	Changes (%)	P-value	Effect size (95% CI)
Avg force left side (N)	0.8 m/s	162 ± 35	169 ± 38	6.9 (−8.8–22.7)	4.2 ± 7.3	1.000	0.55 (-0.4–1.5)
Avg force right side (N)	0.8 m/s	166 ± 42	167 ± 39	0.6 (−13.9–15.1)	0.8 ± 6.0	1.000	0.49 (−0.8–0.9)
Avg force left side (N)	1.5 m/s	141 ± 29	145 ± 34	3.9 (−4.5–12.3)	2.4 ± 3.8	1.000	0.57 (−0.4–1.5)
Avg force right side (N)	1.5 m/s	137 ± 30	143 ± 32	6.2 (−0.8–13.4)	4.5 ± 4.2	0.350	0.87 (−0.4–1.8)
Avg power left side (W)	0.8 m/s	128 ± 28	133 ± 31	5.5 (−7.5–18.5)	4.2 ± 7.6	1.000	0.52 (−0.4–1.4)
Avg power right side (W)	0.8 m/s	131 ± 34	132 ± 31	0.7 (−11.6–13.0)	1.0 ± 6.5	1.000	0.07 (−0.8–0.9)
Avg power left side (W)	1.5 m/s	205 ± 47	213 ± 54	7.9 (−4.4–20.3)	3.5 ± 3.6	0.745	0.80 (−0.3–1.8)
Avg power right side (W)	1.5 m/s	198 ± 46	209 ± 50	10.8 (−0.3–21.8)	5.2 ± 4.3	0.270	0.97 (−0.4–1.9)
Peak acceleration left side (m/s2)	0.8 m/s	18.2 ± 2.5	17.3 ± 4.5	0.9 (−6.2–4.4)	4.8 ± 23.5	1.000	0.21 (−1.1 -0.7)
Peak acceleration right side (m/s2)	0.8 m/s	19.4 ± 2.1	21.3 ± 7.3	1.9 (−5.7–9.6)	8.9 ± 30.2	1.000	0.31 (−0.6–1.2)
Peak acceleration left side (m/s2)	1.5 m/s	23.7 ± 4.0	26.0 ± 7.1	2.3 (−2.0–6.7)	8.6 ± 13.9	1.000	0.67 (−0.3–1.6)
Peak acceleration right side (m/s2)	1.5 m/s	23.6 ± 1.9	26.3 ± 4.6	2.8 (−3.5–9.1)	12.5 ± 22.0	1.000	0.55 (−0.4–1.5)
Peak force left side (N)	0.8 m/s	256 ± 42	281 ± 69	25.0 (−20.9–70.8)	8.9 ± 12.9	1.000	0.68 (−0.3–1.6)
Peak force right side (N)	0.8 m/s	274 ± 67	283 ± 67	9.4 (−9.0–27.8)	3.7 ± 6.3	1.000	0.64 (−0.4–1.6)
Peak force left side (N)	1.5 m/s	250 ± 59	275 ± 69	25.4 (−5.4–56.1)	10.3 ± 12.0	0.420	0.39 (−0.4–1.3)
Peak force right side (N)	1.5 m/s	247 ± 39	256 ± 39	9.3 (−20.2–38.9)	4.2 ± 10.5	1.000	0.39 (−0.5–1.3)
Peak power left side (W)	0.8 m/s	217 ± 31	231 ± 60	14.4 (−30.8–59.7)	5.6 ± 15.6	1.000	0.40 (−0.5–1.3)
Peak power right side (W)	0.8 m/s	229 ± 60	234 ± 56	5.3 (−23.6–34.1)	3.0 ± 10.8	1.000	0.23 (−0.7–1.1)
Peak power left side (W)	1.5 m/s	387 ± 99	428 ± 111	41.4 (−14.8–97.7)	11.3 ± 15.0	0.555	0.91 (−0.2–1.9)
Peak power right side (W)	1.5 m/s	389 ± 64	394 ± 53	4.3 (−49.8–58.5)	1.9 ± 11.8	1.000	0.10 (−0.8–1.0)

* ES, effect size, m = meter, s = second, N = newton, W = watt, CI, confidence interval.

**TABLE 2:** Overview of maximal stroke force and power for the swimmers (n = 12).

Test	Condition	Pre	Post	Mean difference (95% CI)	Changes (%)	P-value	Effect size (95% CI)
Avg force left side (N)	0.8 m/s	193 ± 58	196 ± 56	3.0 (−2.9 -8.9)	2.1 ± 5.3	1.000	0.33 (−0.3–0.9)
Avg force right side (N)	0.8 m/s	199 ± 59	202 ± 63	3.3 (−5.5–12.1)	1.4 ± 7.6	1.000	0.24 (−0.3–0.8)
Avg force left side (N)	1.5 m/s	159 ± 46	163 ± 50	3.8 (−1.4–8.9)	2.0 ± 4.6	0.670	0.47 (−0.1–1.1)
Avg force right side (N)	1.5 m/s	162 ± 48	168 ± 53	5.3 (−3.2–13.9)	2.7 ± 9.0	0.990	0.40 (−0.2–1.0)
Avg power left side (W)	0.8 m/s	152 ± 47	155 ± 46	3.5 (−1.4–8.4)	2.8 ± 5.6	0.730	0.45 (−0.2–1.0)
Avg power right side (W)	0.8 m/s	157 ± 48	160 ± 51	2.8 (−4.6–10.4)	1.6 ± 8.2	1.000	0.25 (−0.3–0.8)
Avg power left side (W)	1.5 m/s	230 ± 72	237 ± 79	7.3 (−1.6–16.2)	2.7 ± 5.6	0.485	0.52 (−0.1–1.6)
Avg power right side (W)	1.5 m/s	235 ± 76	244 ± 84	9.6 (−3.9–23.1)	2.9 ± 4.6	0.730	0.45 (−0.2–1.0)
Peak acceleration left side (m/s2)	0.8 m/s	15.93 ± 6.11	19.38 ± 5.46	3.4 (−1.0–5.9)	27.3 ± 26.8	0.050	0.89 (−0.2–1.6)
Peak acceleration right side (m/s2)	0.8 m/s	15.08 ± 5.33	18.31 ± 5.93	3.2 (−0.1–6.4)	26.4 ± 40.4	0.225	0.65 (−0.1–1.3)
Peak acceleration left side (m/s2)	1.5 m/s	22.66 ± 7.13	23.97 ± 8.66	1.3 (−0.3–2.9)	4.7 ± 11.5	0.470	0.53 (−0.1–1.1)
Peak acceleration right side (m/s2)	1.5 m/s	20.72 ± 6.11	23.50 ± 8.86	2.8 (−0.3–5.9)	11.3 ± 23.9	0.365	0.57 (−0.1–1.2)
Peak force left side (N)	0.8 m/s	263 ± 78	273 ± 86	10.0 (−6.4–26.5)	3.7 ± 9.7	1.000	0.39 (−0.2–1.0)
Peak force right side (N)	0.8 m/s	272 ± 84	280 ± 86	8.0 (−8.3–24.3)	3.2 ± 9.5	1.000	0.31 (−0.3–0.9)
Peak force left side (N)	1.5 m/s	249 ± 77	261 ± 87	4.6 (−4.6–13.8)	4.4 ± 13.7	1.000	0.32 (−0.3–0.9)
Peak force right side (N)	1.5 m/s	241 ± 73	255 ± 89	13.2 (−3.2–29.5)	4.1 ± 12.2	0.520	0.51 (−0.1–1.1)
Peak power left side (W)	0.8 m/s	220 ± 78	249 ± 97	26.0 (−5.2–57.3)	2.8 ± 5.6	0.465	0.53 (−0.1–1.1)
Peak power right side (W)	0.8 m/s	157 ± 48	160 ± 51	8.2 (−17.1–33.5)	1.6 ± 8.1	1.000	0.21 (−0.4–0.7)
Peak power left side (W)	1.5 m/s	230 ± 72	238 ± 79	20.6 (−5.7–47.0)	2.7 ± 5.6	0.560	0.49 (−0.1–1.1)
Peak power right side (W)	1.5 m/s	235 ± 76	244 ± 84	30.4 (−0.8–61.8)	3.3 ± 9.9	0.275	0.62 (−0.0–1.2)

* ES, effect size, m = meter, s = second, N = newton, W = watt, CI, confidence interval.

The isokinetic measures of the maximal stroke test are presented in Table 3. For the 0.8 m/s and 1.5 m/s test velocities, there were no significant differences in average and peak MIF, MIP, and in acceleration, comparing pre- and post-tests (P = 0.095 to 1.000, ES = 0.19–0.63, Table 3). There were no significant effects of sports background or sex on relative changes for any of the isokinetic outcomes (P = 0.176–0.923).

**TABLE 3:** Overview of maximal stroke force and power among all athletes (n = 18).

Test	Condition	Pre	Post	Mean difference (95% CI)	Changes (%)	P-value	ES (95% CI)
Avg force left side (N)	0.8 m/s	184 ± 53	188 ± 52	4.2 (−1.0–9.4)	2.7 ± 5.8	0.545	0.41 (−0.1–0.9)
Avg force right side (N)	0.8 m/s	189 ± 56	192 ± 58	2.5 (−4.2–9.1)	1.2 ± 7.0	1.000	0.19 (−0.3–0.7)
Avg force left side (N)	1.5 m/s	154 ± 41	158 ± 45	3.8 (−0.1–7.7)	2.1 ± 4.3	0.265	0.51 (−0.0–1.0)
Avg force right side (N)	1.5 m/s	155 ± 44	160 ± 48	5.6 (−0.3–11.6)	3.2 ± 7.8	0.310	0.49 (−0.0–1.0)
Avg power left side (W)	0.8 m/s	145 ± 43	149 ± 42	4.1 (−0.2–8.3)	3.2 ± 6.0	0.305	0.49 (−0.0–1.0)
Avg power right side (W)	0.8 m/s	149 ± 45	152 ± 47	2.2 (−3.4–7.9)	1.4 ± 7.5	1.000	0.20 (−0.3–0.7)
Avg power left side (W)	1.5 m/s	223 ± 65	230 ± 72	7.5 (−1.0–14.0)	2.9 ± 5.0	0.130	0.60 (−0.1–1.1)
Avg power right side (W)	1.5 m/s	224 ± 69	234 ± 76	9.9 (−0.6–19.3)	3.8 ± 8.6	0.195	0.55 (−0.0–1.0)
Peak acceleration left side (m/s^2^)	0.8 m/s	16.59 ± 5.32	18.76 ± 5.16	2.2 (−0.1–4.4)	17.8 ± 29.3	0.285	0.50 (−0.0–1.0)
Peak acceleration right side (m/s^2^)	0.8 m/s	16.35 ± 4.97	19.20 ± 6.28	2.8 (−0.2–5.5)	21.3 ± 37.7	0.185	0.55 (−0.0–1.0)
Peak acceleration left side (m/s^2^)	1.5 m/s	22.97 ± 6.27	24.58 ± 8.06	1.6 (−0.2–3.0)	5.8 ± 11.9	0.135	0.59 (−0.1–1.1)
Peak acceleration right side (m/s^2^)	1.5 m/s	21.56 ± 5.33	24.34 ± 7.82	2.8 (−0.3–5.2)	11.6 ± 22.7	0.140	0.59 (−0.1–1.1)
Peak force left side (N)	0.8 m/s	261 ± 68	276 ± 79	14.4 (−0.6–29.4)	5.2 ± 10.6	0.290	0.49 (−0.0–1.0)
Peak force right side (N)	0.8 m/s	272 ± 77	281 ± 79	8.4 (−3.2–20.0)	3.3 ± 8.5	0.710	0.37 (−0.1–0.9)
Peak force left side (N)	1.5 m/s	249 ± 70	260 ± 79	10.7 (−0.5–20.9)	3.8 ± 9.4	0.205	0.54 (−0.0–1.0)
Peak force right side (N)	1.5 m/s	243 ± 64	255 ± 76	12.0 (−0.5–24.6)	4.1 ± 11.4	0.300	0.49 (−0.0–1.0)
Peak power left side (W)	0.8 m/s	220 ± 65	242 ± 87	22.6 (−0.5–45.8)	9.4 ± 17.1	0.270	0.50 (−0.0–1.0)
Peak power right side (W)	0.8 m/s	229 ± 78	236 ± 73	7.4 (−10.7–25.4)	3.3 ± 8.5	1.000	0.21 (−0.3–0.7)
Peak power left side (W)	1.5 m/s	388 ± 123	415 ± 151	26.7 (−5.0–48.5)	5.6 ± 11.4	0.095	0.63 (−0.1–1.1)
Peak power right side (W)	1.5 m/s	379 ± 115	402 ± 138	22.8 (−1.9–47.4)	5.1 ± 13.3	0.340	0.48 (−0.0–1.0)

* ES, effect size, m = meter, s = second, N = newton, W = watt, CI, confidence interval.

**FIGURE 2:**
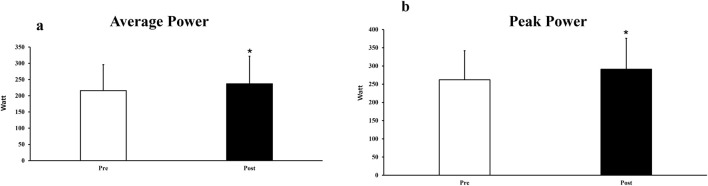
The average power **(a)** and peak power **(b)** pre- and post intervention. * significant difference (p < 0.05) between pre-and post.

**FIGURE 3:**
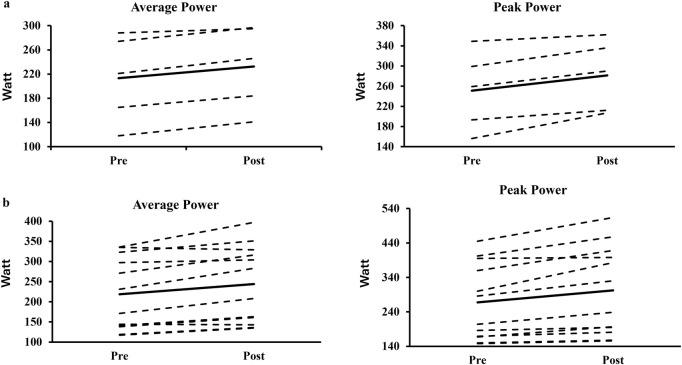
The individual responses of the 20-s sprint test on average power and peak power among the kayak sprinters **(a)** and swimmers **(b)**.

**FIGURE 4:**
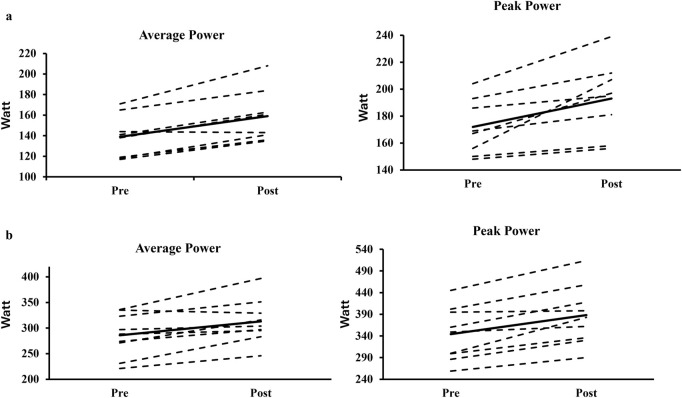
The individual responses of the 20-s sprint test on average power and peak power among the females **(a)** and males **(b)**.

For the 20-s all-out stroke test, AP_20_ significantly improved by 11.9% ± 7.6% (P < 0.001, 217 ± 82 vs 241 ± 87 W, *ES* = 0.28, Figure 2a) and PP_20_ significantly improved by 12.8% ± 8.4% (P < 0.001, 263 ± 100 vs 296 ± 113W, ES = 0.30, Figure 2b). The factors ‘sex’ and ‘sports background’ did not significantly affect relative changes in AP_20_ (P = 0.243–0.668) or PP_20_ (*p* = 0.901 to 0.691). For individual training responses across sport background and sex, please see Figures 3, 4.

## Discussion

The main findings of the present study were that 8 weeks of HR-CST significantly improved 20-s all-out stroke performance in national-level junior athletes, irrespective of sports background (i.e., kayak sprinters vs swimmers) or sex (i.e., females vs males). Performance outcomes examined in the maximal stroke test (i.e., peak/average MIF, MIP, acceleration) were not significantly affected following HR-CST in these junior athletes.

Initially, we hypothesized that maximal and 20-s all-out stroke test performance would improve following HR-CST in national-level junior athletes, due to potential improvement in trunk muscle inter-coordination and co-contraction capacity, leading to increased trunk stability and enhanced force transfer. However, the present findings only partly supported the hypothesis. More specifically, only AP_20_ and PP_20_ improved by 11.9% and 12.8% in all athletes, respectively. It could be speculated that a short-term learning effect (especially for the swimmers) might have occurred contributing to the observed significant performance gains however, the kayak sprinters within the participant group were already familiarized with the paddle ergometer device, due to its training-related specificity. Furthermore, relative changes across time were similar for the kayak sprinters and the swimmers and therefore, we speculate that the performance gains observed at post-test could primarily be attributed to HR-CST. According to this hypothesis, improvements observed could represent a dose-response effect for this exercise-regime, which targets universal mechanisms of force transfer and force production. Another potential explanation might be that some of the exercises un-intentionally mimicked the relevant motions of both sports and, in support of this, evidence from baseline tests showed no effect of sports background However, the approach of the training-paradigm used in this study utilized universal mechanisms of force production and transfer, through dynamic core strengthening exercises at high loads and few repetitions, unstable surfaces, and unilateral loading, with a rotational component. Importantly, the observed effect sizes were small (ES = 0.28–0.30), which should be taken into account, when interpreting results, along with lack of inclusion of a control group, which is a further limiting factor. It is also possible that the lack of familiarization influenced the performance outcomes, particularly among swimmers or, alternatively, that the improvements observed could be a result of pre-season preparation and training periodization. Nevertheless, when examining the individual pre-post responses (Figures 3, 4), most of the athletes improved their stroke performance (independent of sex or sports background) with the replacement of 45 min of their traditional strength training twice per week as the only significant change in the athletes’ regular training routines.

Both swimmers and kayak sprinters require a strong and stable trunk to maintain position in the water or boat, while maximizing the force and power during the strokes. The present findings are in alignment with previous comparable studies in swimming (Karpinski et al., 2020; Weston et al., 2015), golf (Sung et al., 2016) and handball (Saeterbakken et al., 2011; Dahl and van den Tillaar, 2021). For example, Karpinski et al. (2020) and Weston et al. (2015) demonstrated a 1.2% and 2.0% improvement in 50m crawl among national-level junior and senior swimmers after 6 weeks (Karpinski et al., 2020) and 12 weeks (Weston et al., 2015) of core training, respectively. Importantly, all these studies conducted a progressive HR-CST trying to mimic specific movement patterns among national-level athletes. The HR-CST training protocol in the present pilot study and other comparable studies (Saeterbakken et al., 2011; Sung et al., 2016; Karpinski et al., 2020; Weston et al., 2015; Dahl and van den Tillaar, 2021), emphasized the principle of overload (Kraemer et al., 1998) (few repetitions, high loads), task-specificity (Behm and Sale, 1993; Saeterbakken et al., 2025) (unilateral and unstable) and mimicking the specific movements (dynamic trunk rotational component) used in traditional RT programming for other muscle groups. In contrast, several of the previous studies applied isometric low-intensity, high-volume core training among athletes (Stanton et al., 2004; Tse et al., 2005; Patil et al., 2014; Schibek, 1999), which may explain why they did not demonstrate improvement in physical/sport-specific performance (Rodríguez-Perea et al., 2023; Saeterbakken et al., 2022; Prieske et al., 2016b) or more specifically, in swimming (Schibek, 1999), running (Stanton et al., 2004), or rowing (Tse et al., 2005). Therefore, it could be speculated that the core strength training program should be designed to mimic the respective sports movements and intensity, in addition to maintaining stability of the lumbopelvic-hip complex, while generating force and power from the upper and lower extremities (i.e., the serape effect). Whether the improvements were caused by 1) greater strength levels of the core, 2) the ability to stabilize the trunk reducing loss of force transfer between segments, or a combination of 1) and 2), cannot be clearly determined for the current pilot study. Of note, tests measuring core strength and/or core stability were not included and should be considered in future studies.

In contrast to our hypotheses, MIF and MIP did not significantly improve, with small to moderate effect sizes (ES 0.48–0.63) observed. As a result of low participant numbers, the power of the study to detect an effect of the core training intervention on these parameters may have occurred (possible type 2 error). Although a paddle stroke in water is not exclusively isokinetic, there are similarities between the tested isokinetic resistance mode and paddle stroke resistance on water (Zinke et al., 2019). Furthermore, and unexpectedly, no improvements in maximal stroke performance were observed for any of the two test velocities (0.8 m/s and 1.5 m/s). Our results are not supported by findings among world-class kayak sprinters (Zinke et al., 2019). Zinke et al. (2019) demonstrated increased isokinetic trunk rotation torque after an 8-week progressive isokinetic trunk training period. Furthermore, a strong relationship was observed between the isokinetic torque and peak paddle force (Zinke et al., 2019) which possibly could explain the findings of the 20-s kayak sprint test, but not the findings of the MIF and MIP. Importantly, future studies should include reliable and validated tests of core strength and stability pre- and post HS-CST interventions, to examine whether changes in core strength demonstrate a ‘transfer effect’ in relevant metrics that quantify sports-specific actions. Future studies should therefore expand the sample size, include a control group, examine different athletic populations and include familiarization of the testing- and training protocols. The Supplementary Material includes detailed information about the exercises, such as execution and progression. We hope future studies may use these and previous insights/findings (Saeterbakken et al., 2011; Sung et al., 2016; Karpinski et al., 2020; Weston et al., 2015; Dahl and van den Tillaar, 2021) as guidance when developing core training protocols for athletes.

The present study is a pilot study and has several limitations, which need to be acknowledged. First, the study did not include a control condition and we cannot conclude whether learning effects, HR-CST, regular training, or a combination of the aforementioned variables contributed to the improved 20-s stroke test performance in national-level junior athletes. The present findings can be considered as preliminary and must, therefore, be interpreted with caution. Secondly, the sample size was rather small and may have compromised statistical power. However, recruiting high numbers of higher-level junior athletes, particularly in individual sports, is challenging, as indicated in previous studies (Karpinski et al., 2020; Ika et al., 2022; Weston et al., 2015). Therefore, the sample size was a result of convenience. Thirdly, we included an upper-body strength and power test that was specific for kayak sprinting but not swimming (e.g., 50-m crawl), although the stroke tests partly mimicked the movement patterns of swimming (i.e., unilateral strokes with trunk rotation). Fourthly, test reliability of the 20-s all-out stroke tests was not undertaken, which limits the potential to exclude possible short-term learning effects for the tests. However, test reliability between the two strokes in the isokinetic testing demonstrated excellent ICC (i.e., >0.90). Finally, we did not include a core strength/stability test, and the effects of the intervention can only be quantified by the instructors’ observation (i.e., ability to perform the exercises correctly and progress them) during the intervention. Despite this limitation, the core strengthening exercises used were progressed either in resistance and difficulty, or complexity (please see Supplementary Material). Progression was therefore gradual and increased on an individual basis throughout the intervention period, which in itself demonstrates an improvement effect across the intervention period.

## Conclusion

As one of the first studies to do this, we examined the effects of 8-week of 45 min progressive high-load, dynamic core training (HR-CST) twice per week in mixed-sex, multi-sport national level junior athletes. The swimmers and kayak sprinters improved performance in a 20-s kayak sprint test and achieved greater peak acceleration during a maximal stroke action, with similar training responses observed independent of sport-specific training background and biological sex. Hence, HR-CST seems to be a viable option for junior athletes wanting to improve high force upper limb actions and, specifically, paddling performance.

## Perspectives

In the present pilot study we tried to design a progressive HR-CST program that mimicked stroke patterns during kayak sprinting and swimming, and at higher intensities during dynamic movement, in addition to the focus on core competence/stability (i.e., capacity to maintain iliac crest alignment in multiple planes, avoiding trunk sway and lumbopelvic tilt). Furthermore, several sports (throwing, kicking, swimming and kayak sprint) involve rotations along the vertical axis. Therefore, designing effective three-dimensional core strengthening exercises is relevant to performance and is in accordance with the principle of training specificity. In the present study, we designed a series of dynamic core exercises featuring rotational elements and unilateral movements performed in unstable conditions. Coaches and practitioners should explore the application of these movements but importantly, gradually increase intensity when the athletes master the basic exercise actions. We strongly emphasize the importance of high movement quality, i.e., maintaining stability and control in the core, and not progressing intensity, complexity or stability demands before the athletes have gained a foundation in movement quality. Among beginners or young athletes, we recommend a higher number of repetitions at lower intensity (higher training volume) in accordance with RT recommendations. Having a highly experienced instructor supervising the intervention, to correct and identify deficits in movement quality, should be considered important, as movement quality is key during core strengthening exercises, irrespective of whether the aim of the exercise is to improve stability, strength or muscular endurance.

## Data Availability

The original contributions presented in the study are included in the article/Supplementary Material, further inquiries can be directed to the corresponding author.
