# Supplementary material for: The effect of heavy-resistance core strength training on upper-body strength and power performance in national-level junior athletes–a pilot study
Source: Front Physiol. 2025 Jun 11;16:1617104. doi: 10.3389/fphys.2025.1617104 (PMC12187601; doi:10.3389/fphys.2025.1617104)
Supplement: Supplementary file 1 [file DataSheet1.pdf]

Supplementary file: Training procedures

Each core training session was two-phased and consisted of a 5–8-minute warm-up session with 4 to 5 exercises, and a main session of 4 – 6 exercises. The warm-up included one set of 10-12 repetitions of lower load exercises using a Swiss ball or suspension training equipment, requiring slow and controlled movements. The warm-up exercises were designed to engage core muscles and included both balance and stability exercises. For the main session, 12 high-resistance exercises were designed (please see further details in the table below). Several of the exercises target the same muscles and these exercises were rotated throughout the intervention period. Furthermore, and based on the previous mentioned programming to target core capacity [1], the exercises had different progression levels in either resistance, difficulty or movement complexity [2]. The progression was therefore gradual, and increased on an individual basis throughout the intervention period [3]. Both the warm-up and main session included a focus on core stability, awareness and control of lumbopelvic position, as well as core strength.

In general, the level of difficulty and load was increased continuously on an individual basis, as soon as indicated by the athlete`s readiness and mastery level [2]. The athletes were encouraged to increase the level of difficulty and load gradually for each exercise and session, as their technical performance of the exercises progressed, in accordance with general principles of loading [4]. The intensity gradually increased as strength and motor control in these exercises were developed, until they could complete the whole exercise with maximal effort.

The level of difficulty can be progressed by increasing lever arm (higher loading demand), instability of exercise surface, or by reducing base of support [5], and progression from bilateral to unilateral loading on exercises that allow this [6], adding rotational components/transverse plane loading bias [7]. The athletes were also told to maintain a stable core through the exercises, by means of either avoiding increased lumbar lordosis (hyper-lordosis), or by flattening of lumbar lordosis hypo-lordosis) prior to increased loading of the spine.

An overview of the exercises in the main session and the progression

	Exercises main session	Progression
1	Supine spinal rotation in slings. One leg in large sling, rotate opposite leg and pelvis up until the free leg touches the sling, keep rotating as far as possible pushing free leg into the outside top of sling and back down. Focus on slight flattening of lumbar lordosis to avoid hyper-extension.	With hands up: sling placement under calf muscles, move sling towards ankle, increase distance between ankles. With arms to the side: same rotational movement with Swiss ball held between ankles.
2	Standing: forward leaning spinal rotation in sling. Large sling over chest and upper arms, hands on the outside, holding on to top of the sling. Plantar flexion of ankles. Twisting movement with upper body, emphasize low impact on lift off and landing.	Move feet backwards to increase body inclination (forward lean angle).
3	Iliopsoas / Sandvikmoen. Flattening of low back with incremental load in supine	Push low back down and hold for 1 minutex3. Push low back down and hold

	lying in sling: Supine lying with low back and pelvis suspended in large sling 10 cm above floor. Hips and knees at right angles, thighs in parallel with ropes.	for 3 secs, push front of thighs against ropes, relax. Repeat the above, during the 3 secs push thighs harder into ropes until upper body lifts-off. Repeat the above, add twisting movement while in the air. Repeat the above on one leg.
4	Prone frontal plane loading in 3 positions in slings: 1.Superman, adopt deep squatting position, slings in hands, lean forward as legs straighten and shoulders are flexed until whole body straightens, return to start. 2.Reversed dynamic bridge: standing with knees straight and hips and back flexed, hands in slings in front of body, lean forward until whole body is straight, slow execution in both directions. 3.Full frontal horizontal body flexion, handstand with straight body, feet in large sling, start flexing hips with straight knees, bring feet as close towards arms as possible, slow execution in both directions.	Height of smaller slings 5 cm above floor, can be higher if needed for reduced challenge.<br>1.Superman: move feet further back to increase body incline (angle of lean). 2. Reversed dynamic bridge: moving feet further back will give increased pull from ropes, adjust until correct load is achieved. 3. Same principle as above: move hands forward on floor.
5	Prone parachuting / sky diving in large sling, fully suspended: Fold a large sling into two, place on the front of pelvis (ASIS), take a fully flexed position with feet and hands off the floor, slowly lift arms and feet until whole body is straight, stabilize for 3 to 5 seconds, return. Slow and controlled execution.	If low back allows, lift into hyper-extension. Note: not appropriate for some individuals to hyper-extend the spine. Swiss balls or other light weights can be added to hold between feet and between hands.
6	Backward leaning one-arm pull-up in sling: Standing: lean back with one hand through smaller sling, feet together. Lean backward and rotate, bend knees, free elbow reaches to floor, return and continue rotating until free elbow reaches as high on the rope as possible.	Use one arm and opposite leg, same rotational pull-up movement. Pull towards hip. Standing diagonal pullup. Horizontal explosive pull-up.
7	Prone bridge in sling. «Sterk som Bjørgen»: Elbows on floor, one leg in large sling, free leg in the air in parallel. Use shoulder movement to shift the whole body forward until forehead touches the floor, then shift backwards until head touches floor again, producing a rocking movement backwards and forwards.	Copy the backward shift, then during forward shift move smoothly from elbow to handstand position and pull knees to chest, return and repeat. Can be performed in diagonal loading pattern on one arm only. Can be done fully suspended, with smaller slings in hands and one large sling on one leg only. Extend whole body, then bring knees and hands to touch, repeat.
8	Prone lying spinal rotation in large sling: Lying on elbows, one leg in wide sling, the free leg outside the sling on the	Start with the sling under thigh or knee, gradually move sling down to ankle or foot, as tolerated. Progress by adding

	«opposite» side, giving a rotated starting position, feet together. Rotate lower body against the momentum of the slings, producing a coiling effect where the lower body is slightly elevated, return to full rotation to opposite side, aiming for 180 degrees measured at the feet.	whole body anterior (sagittal) flexion, bringing knees towards chest during rotation = «corkscrew» spiral movement.
9	«Thread the needle» (tre nåla) on the outside of ropes: Starting position; hanging by the arms in smaller slings or with larger sling under armpits/upper back. Start by slightly flattening the lumbar lordosis, then perform whole body flexion by slowly lifting legs and feet to the ropes or slightly beyond them.	Progress from larger sling under upper back, to smaller slings hanging by the arms. Place one leg on the outside of the rope, lock the body in the high position for at least 1 second. Bring both legs to the outside of the rope, lock for 3 seconds.
10	Hanging clock / «around the world». Suspended supine spinal rotation: Starting position; hanging by the arms in smaller slings or with larger sling under armpits/upper back.	Start with larger sling under armpits/upper back, raise locked feet towards ceiling until legs are in parallel with ropes=legs at 12 o`clock. Bring legs to 11 then 1 o`clock, then 10 and 2 etc.<br>Progress to hanging by the arms in smaller slings, repeat the above to perform full circles from 6 to 6 o`clock.
11	«Starfish» Sidelying bridging, legs in large sling: Lift pelvis off floor until body reaches a slightly arched position upwards, at the same time lift upper leg and upper arm towards ceiling.	Start with lying on the shoulders, sling placed at knees, progress by moving sling towards feet. Progress to lying on elbow, then to one arm handstand, performing the same movement. Can be further progressed by adding «scissor» movement with legs; lower leg is pushed c into sl into sling, upper leg pushed forward while in upper position of the exercise, to increase activation of glutes.
12	One-arm crunch in large sling: Starting position sidelying with lower leg in sling, free leg on the back of ropes/sling, feet in locked position, erect body posture with pelvis off the floor. The free hand is held by the hip, without touching it.	From starting position do a full body Trunk (sagittal) flexion, bringing knees to chest. Progress by adding rotation of body and the weightbearing shoulder, bringing body into prone position while fully flexed. Decrease load by moving elbow placement closer to point of suspension in starting position, increase load by moving elbow further away.

References

1. Kibler, W.B., J. Press, and A. Sciascia, *The role of core stability in athletic function.* Sports Med, 2006. **36**(3): p. 189-98.
2. Saeterbakken, A.H., R. van den Tillaar, and S. Seiler, *Effect of core stability training on throwing velocity in female handball players.* J Strength Cond Res, 2011. **25**(3): p. 712-8.
3. Saeterbakken, A.H., et al., *Effects of ten weeks dynamic or isometric core training on climbing performance among highly trained climbers.* PLoS One, 2018. **13**(10): p. e0203766.
4. Hibbs, A.E., et al., *Optimizing performance by improving core stability and core strength.* Sports Med, 2008. **38**(12): p. 995-1008.
5. Saeterbakken, A., et al., *The Effect of Performing Bi- and Unilateral Row Exercises on Core Muscle Activation.* Int J Sports Med, 2015. **36**(11): p. 900-5.
6. Saeterbakken, A.H. and M.S. Fimland, *Muscle activity of the core during bilateral, unilateral, seated and standing resistance exercise.* Eur J Appl Physiol, 2012. **112**(5): p. 1671-8.
7. Dahl, K.S. and R. van den Tillaar, *The Effect of Eight Weeks of Sling-Based Training with Rotational Core Exercises on Ball Velocity in Female Team Handball Players.* J Hum Kinet, 2021. **77**: p. 261-272.
